# Supplementary material for: Life without sex: Large-scale study links sexlessness to physical, cognitive, and personality traits, socioecological factors, and DNA
Source: Proc Natl Acad Sci U S A. 2025 Sep 16;122(38):e2418257122. doi: 10.1073/pnas.2418257122 (PMC12478097; doi:10.1073/pnas.2418257122)
Supplement: Supplementary file 1 — Appendix 01 (PDF) [file pnas.2418257122.sapp.pdf]

Supplementary Figures

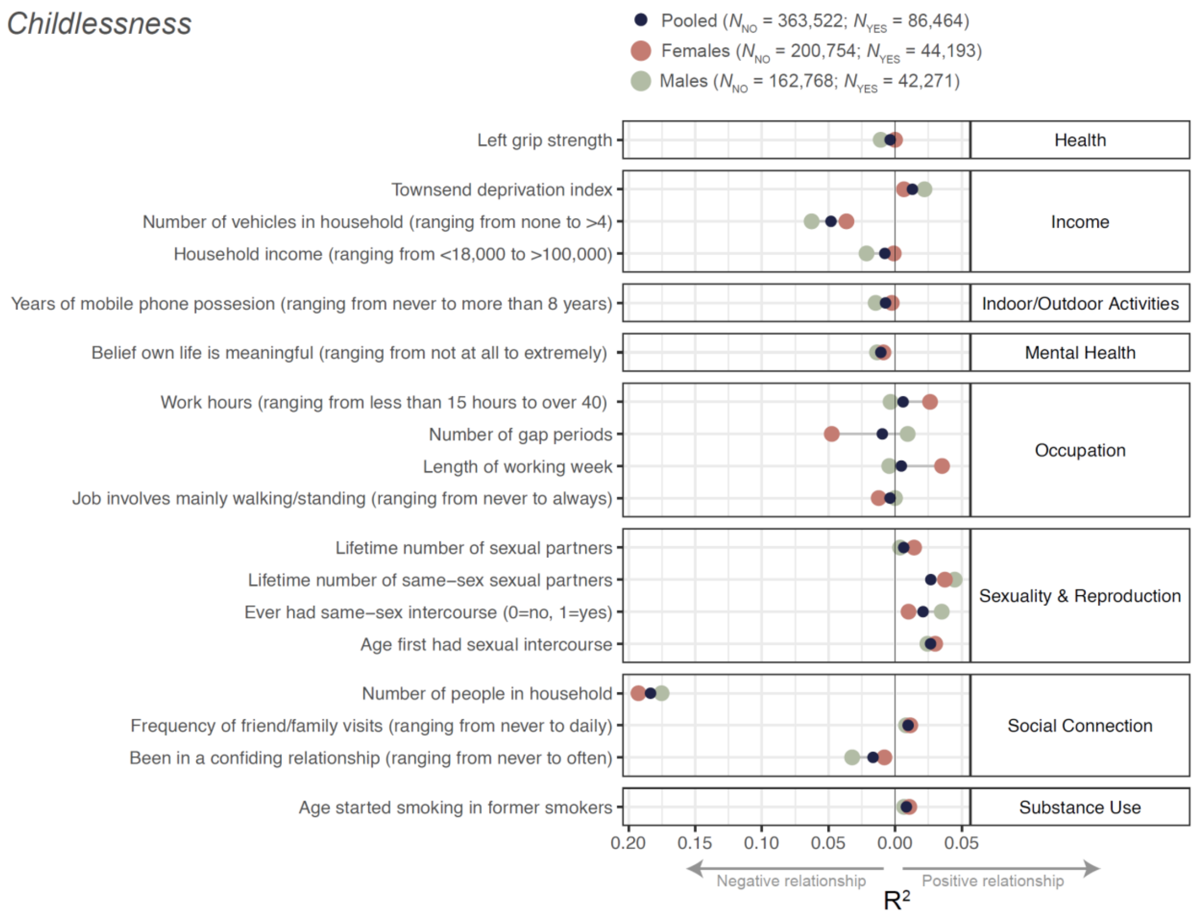

Figure S1: Phenotypic associations of childlessness with health, psychological, and behavioural outcomes. *Childlessness was coded as 0=has children, 1=has no children. Results are shown for the full (sexes pooled) sample, as well as males and females separately. Only associations that were significant in at least one of the analyses (i.e. sexes pooled, males, or females) and explaining 1% or more variance are shown. Full results can be found in Dataset S1 Tab 2. Note that to improve clarity, in some instances variable names and coding have been changed from the original UK Biobank names/coding, see Dataset S1 Tab 1. Note that there were too few female participants who had nonzero numbers of 'lifetime number of same-sex sexual partners', so this estimate is missing.*

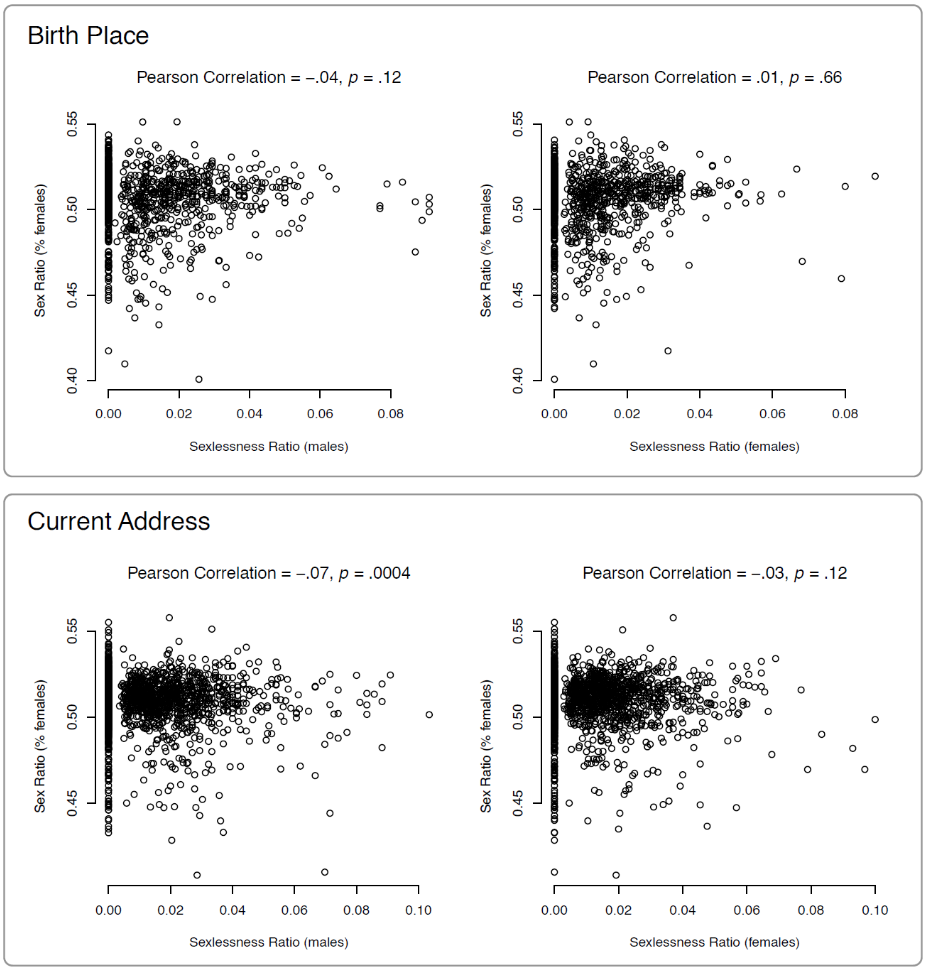

Figure S2: Sex ratio (proportion of women) versus sexlessness ratio, for birth place and current address. *Sex ratio was based on census data and sexlessness ratio on UK Biobank data. Top panel: the Figures show results for 2,476 MSOA regions of the current address with at least 50 UK Biobank participants per region. The average sex ratio is .51. Bottom panel: The Figures show results for 1,334 MSOA regions of the birth place with at least 50 UK Biobank participants per region. The average sex ratio is .51. The average sexlessness ratio is .01 for males, and .01 for females.*

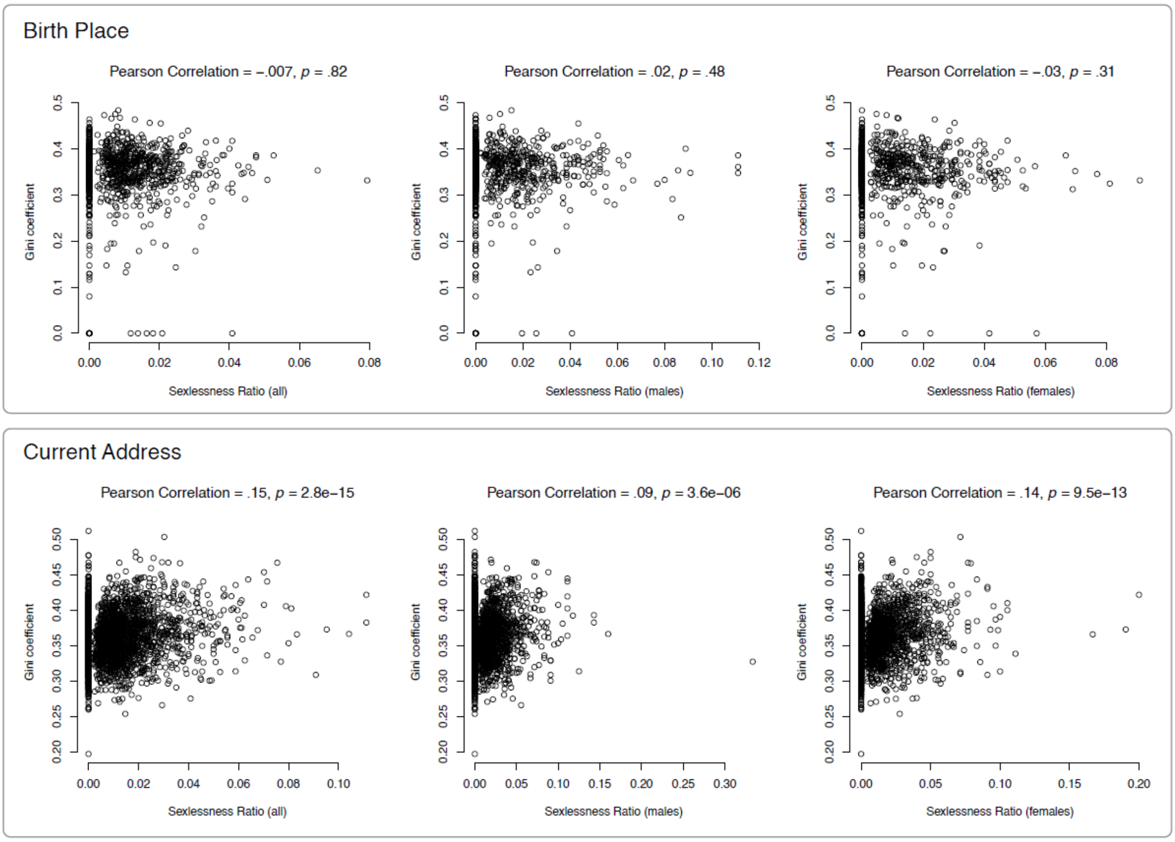

Figure S3: Income inequality versus sexlessness ratio for birth place and current address. *Both income inequality and sexlessness ratio were derived from UK Biobank data. Top panel: the Figures show results for 1,499 MSOA regions of the birth place with at least 50 UK Biobank participants per region. The average GINI coefficient is .35. Bottom panel: The Figures show results for 2782 MSOA regions of the current address with at least 50 UK Biobank participants per region. The average GINI coefficient is .36. The average sexlessness ratio is .01 for the pooled sample, .01 for males, and .01 for females.*

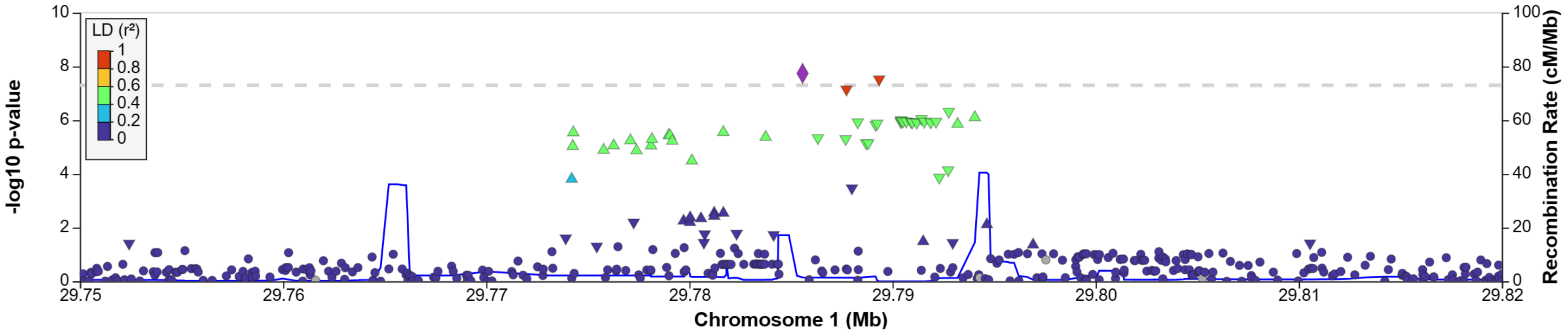

Figure S4: Locus zoom plot of the region with the genome-wide significant SNP.

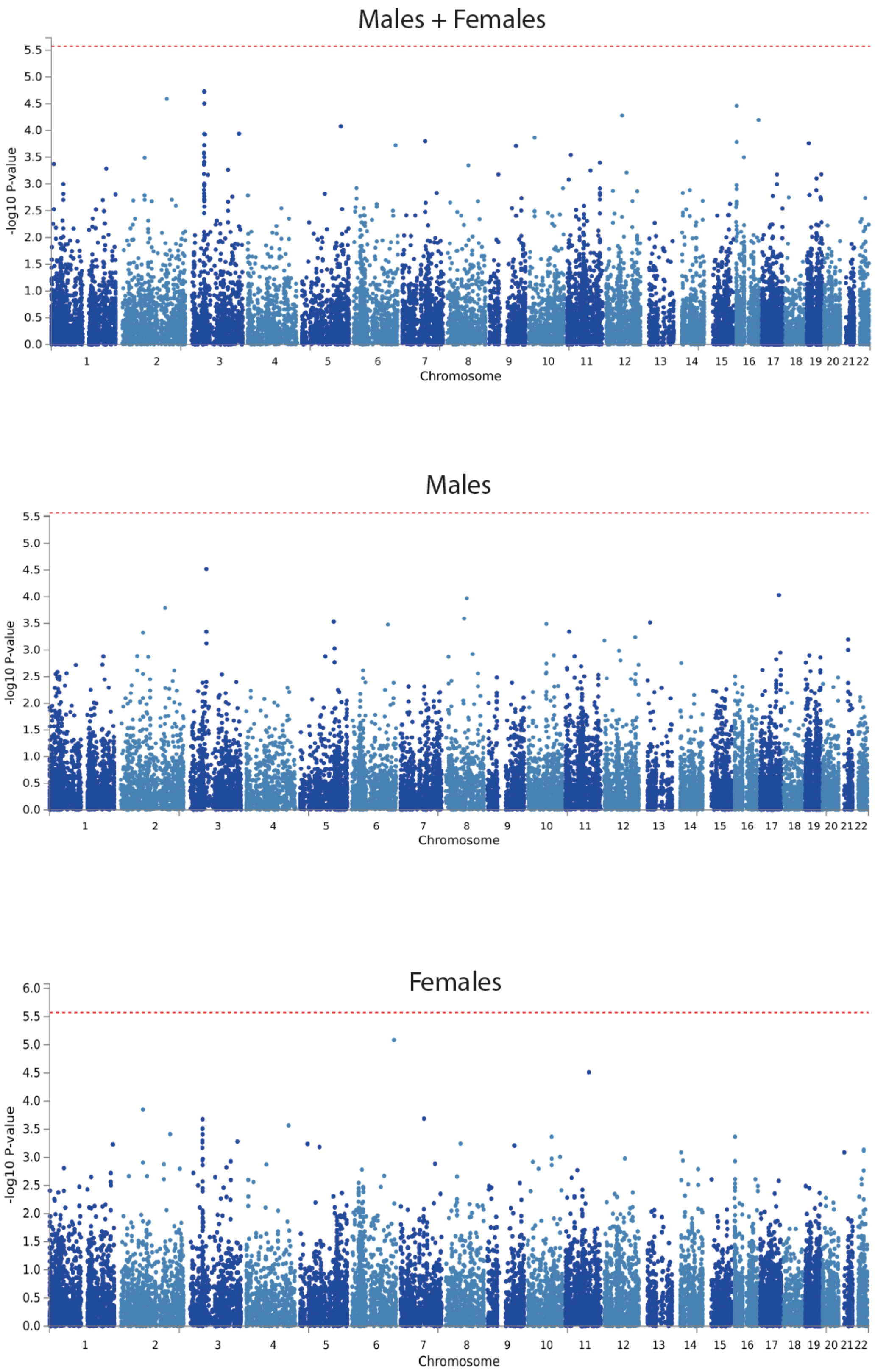

Figure S5: Manhattan plots of the MAGMA gene-based tests for males and females pooled and separately. *The red line indicates the threshold of genome-wide significance.*

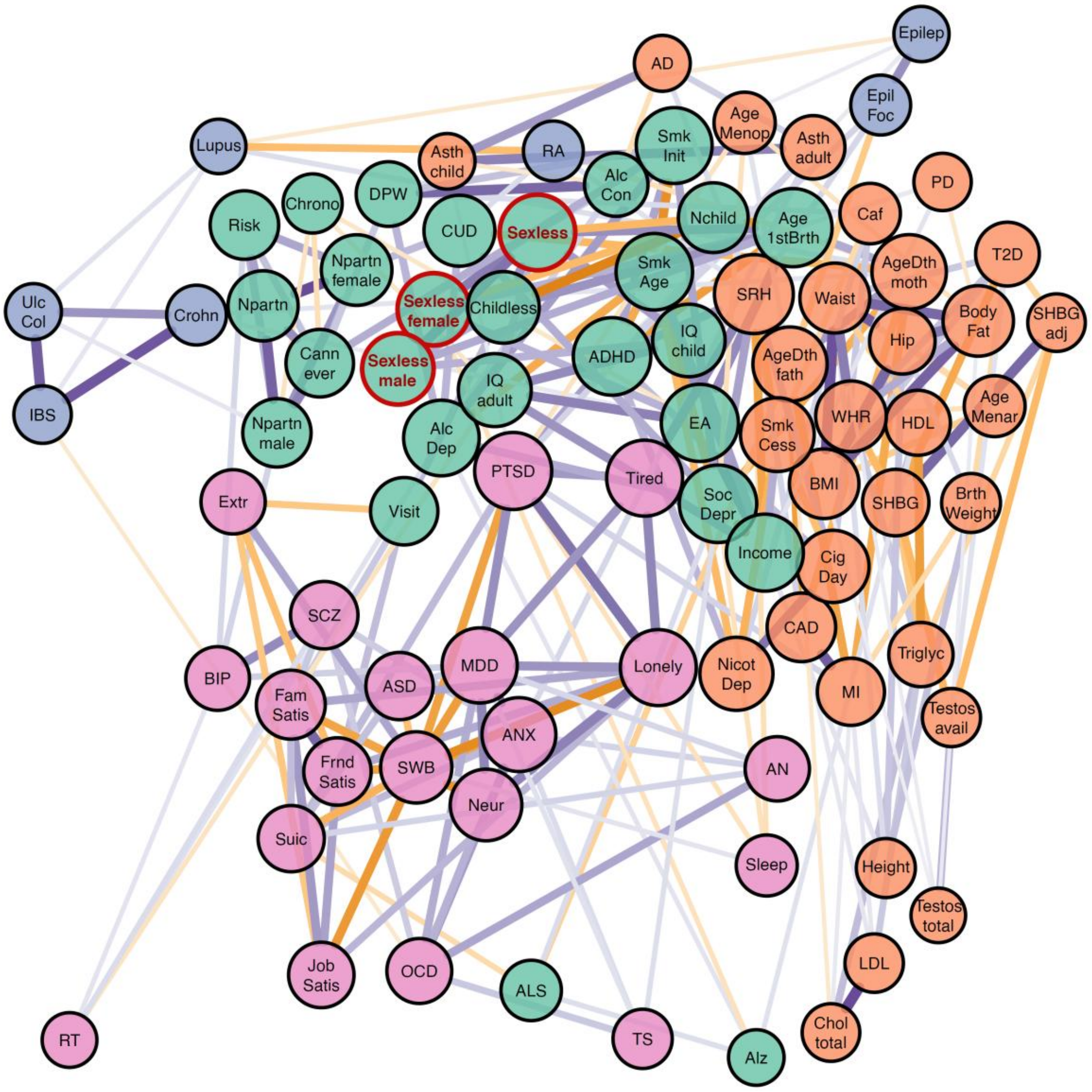

Figure S6: Visualization of the SNP-based genetic correlations between sexlessness and all other traits as a graph. *Each vertex (node) represents a trait, with the colouring denoting different trait groups). Weighted undirected graphs were created with the absolute genetic correlation as connection strength (represented by line thickness). Orange edge colours represent negative genetic correlations and purple edges positive ones. Vertex size is based on eigenvector centrality with a minimum offset. Vertex layout was based on the Früchterman and Reingold algorithm[1]. Colouring is based on a clustering algorithm on the genetic correlation matrix, and reflects what cluster the sexlessness belongs to. Phenotype names corresponding to the abbreviations can be found in Dataset S1 Tab 7.*

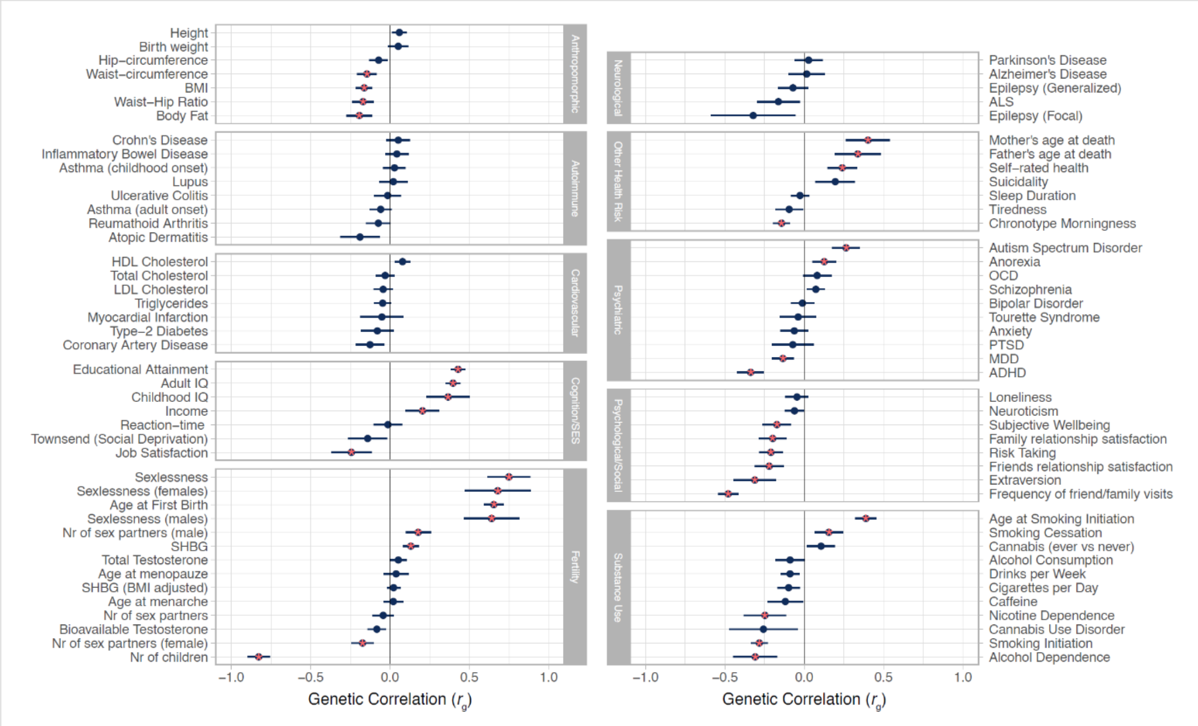

Figure S7: Genetic correlations (r_g) between childlessness and a wide variety of complex traits, computed with LDSC regression.

Supplementary References

1 Fruchterman, T. M. & Reingold, E. M. Graph drawing by force-directed placement. *Software: Practice and experience* **21**, 1129-1164 (1991).
